# Supplementary material for: Content-rich biological network constructed by mining PubMed abstracts
Source: BMC Bioinformatics. 2004 Oct 8;5:147. doi: 10.1186/1471-2105-5-147 (PMC528731; doi:10.1186/1471-2105-5-147)
Supplement: Additional File 2 — The original results of the above study (non-essential files are deleted to keep the file size under the limit set by BMC bioinformatics). [file 1471-2105-5-147-S2.bz2 › chilibotAdditionalFile2/dip50/384ID1375038E9797/html/NTRK2_BDNF.html]

 


 **NTRK2** and **BDNF** 
  
Found 857 abstracts in PubMed, retrieved 50.  
 

 What does Google say? 
 PDF only 
| .edu only 

---

**Interactive relationship** (e.g. stimulation, inhibition, etc)

**Stimulatory relationship**- CONCLUSION CSC could salvage the degenerating neurons and maintain their survival after cortical devascularization by increasing the expression of  **BDNF**  and trkB  [ **NTRK2** ]  mRNA and improving the synthesis of  **BDNF**  mRNA and trkB  [ **NTRK2** ]  protein.  Ref: 12575383 Zhongguo Zhong Xi Yi Jie He Za Zhi, 2001
- Moreover, three types of TrkB  [ **NTRK2** ]  dimers were induced by stimulating with exogenous  **BDNF** .  Ref: 12706205 J Chem Neuroanat, 2003
- Lithium treatment time dependently increases the intracellular level of  **BDNF**  in cortical neurons and activates its receptor, TrkB  [ **NTRK2** ] .  Ref: 12701214 Seishin Shinkeigaku Zasshi, 2003
- Recent experimental evidence supports the role of  **BDNF**  in memory processes Memory acquisition and consolidation are associated with an increase in  **BDNF**  mRNA expression and the activation of its receptor TrkB  [ **NTRK2** ] .  Ref: 12719654 J Pharmacol Sci, 2003
- CONCLUSIONS  **BDNF**  quickly increases the release of dopamine from amacrine cells in the rabbit retina by an action that is likely to involve TrkB  [ **NTRK2** ]  receptors.  Ref: 12556415 Invest Ophthalmol Vis Sci, 2003
**Neutral relationship**- Our findings suggest the importance of interaction between  **BDNF**  TrkB  [ **NTRK2** ]  signaling and NMDA receptors for spatial memory in the hippocampus.  Ref: 12663749 Learn Mem< MedlineTA>Learn Mem, 2003
- **BDNF**  binds to the receptor tyrosine kinase TrkB  [ **NTRK2** ]  and transmits neurotrophic signals by activating neuron specific tyrosine phosphorylation pathways.  Ref: 12803487 Mol Cells, 2003
- Furthermore, recent findings suggest the importance of interaction between  **BDNF**  TrkB  [ **NTRK2** ]  signaling and NMDA receptors for spatial memory.  Ref: 12719654 J Pharmacol Sci, 2003
- We conclude that  **BDNF**  modulates GABAA receptor mediated activity through TrkB  [ **NTRK2** ]  receptor signalling that triggers a kinase dependent short latency effect and a delayed longer latency effect hallmarked by receptor internalization.  Ref: 12640011 J Physiol< MedlineTA>J Physiol, 2003
- This indicates that the activation of the TrkB  [ **NTRK2** ]  path through PI 3 kinase is required for  **BDNF**  s survival promoting effects.  Ref: 12691830 Cancer Lett, 2003
- These observations suggest that the functions of  **BDNF**  may be modified by interaction among subtypes of TrkB  [ **NTRK2** ]  in each region of the Purkinje cells.  Ref: 12706205 J Chem Neuroanat, 2003
- The mammalian trkB  [ **NTRK2** ]  locus undergoes alternative splicing to produce two different types of brain derived neurotrophic factor  [ **BDNF** ]  receptors.  Ref: 12654337 Neuroscience, 2003
- **BDNF**  then binds to and activates its cognate receptor, receptor tyrosine kinase B TrkB  [ **NTRK2** ] .  Ref: 12853306 Ann N Y Acad Sci< MedlineTA>Ann N Y Acad Sci, 2003
- The upregulation of  **BDNF**  expression, in turn, produced neuroprotective signals through receptor tyrosine kinase B TrkB  [ **NTRK2** ]  and promoted cell survival under the conditions of oxygen and glucose deprivation.  Ref: 12738784 J Biol Chem< MedlineTA>J Biol Chem, 2003
- Brain derived neurotrophic factor  [ **BDNF** ]   **BDNF** , a neurotrophin, binds to and activates its specific receptor tyrosine kinase, TrkB  [ **NTRK2** ] .  Ref: 12809697 Neuroscience< MedlineTA>Neuroscience, 2003
- Brain derived neurotrophic factor  [ **BDNF** ]  has been shown to modulate synaptic plasticity in the corpus striatum in vitro by activation of the tyrosine kinase linked receptor, TrkB  [ **NTRK2** ] .  Ref: 12644273 Brain Res, 2003
- We blocked  **BDNF**  activation of TrkB  [ **NTRK2** ]  using a dominant negative TrkB  [ **NTRK2** ]  mutant or a TrkB  [ **NTRK2** ]  function blocking antibody, and found that this protected motor neurons against excitotoxic insult in cultures of mixed spinal cord neurons.  Ref: 12614342 J Neurochem, 2003
- When cerebellar cultures were simultaneously exposed to activity blocking agents and to neurotrophins  **BDNF**  or NT 4, both of which bound to the TrkB  [ **NTRK2** ]  receptor, the numbers of inhibitory Purkinje cell axosomatic synapses were similar to those of untreated control cultures, and control rates of spontaneous cortical discharges were recorded.  Ref: 12647813 Histol Histopathol, 2003

**Non-interactive relationship** (e.g. studied together, co-existance, homology, etc.)

- To test this, we infused the cortex with an inhibitor of Trk receptor tyrosine kinases, anti TrkB  [ **NTRK2** ]  IgG1, anti  **BDNF** , and anti neurotrophin 4 5 antibodies.  Ref: 12736347 J Neurosci, 2003
- Our findings suggested that activation of TrkB  [ **NTRK2** ]  PI3 K and protein synthesis signaling pathway by  **BDNF**  in the hippocampus is important for spatial memory.Molecular Psychiatry 17 224.  Ref: 12610654 Mol Psychiatry, 2003
- These data highlight the fact that even mild injury results in sustained changes in nociceptive circuitry and raise the possibility that the brain derived neurotrophic factor  [ **BDNF** ]  TrkB  [ **NTRK2** ]  system may contribute to persistent pain after tooth repair.  Ref: 12597715 J Endod, 2003
- RESULTS Seizures induce a strong upregulation of TrkB  [ **NTRK2** ] , the receptor for brain derived neurotrophic factor  [ **BDNF** ] .  Ref: 12558581 Epilepsia, 2003
- Our results suggest that overexpression of  **BDNF** , NT 3, trkB  [ **NTRK2** ]  and trkC in motoneuron areas neighboring the site of mechanical compression may represent compensatory changes in response to the compromised neuronal function at the level of compression,.  Ref: 12774239 Acta Neuropathol Berl , 2003
- **BDNF**  increased the density of vesicles and deprivation of endogenous TrkB  [ **NTRK2** ]  ligands with TrkB  [ **NTRK2** ]  fusion protein reduced the density of vesicles in the synapses, without effects on synapse number or docked vesicles.  Ref: 12577323 J Comp Neurol, 2003
- In the Schaffer collateral pathway of the hippocampus, binding of  **BDNF**  to high affinity trkB  [ **NTRK2** ]  receptors is essential for the induction of long term potentiation, a specific type of synaptic plasticity.  Ref: 12628355 Trends Pharmacol Sci, 2003
- In the present study, we investigated the neuroprotective effects of acupuncture on the nigrostriatal system in rat unilaterally lesioned with 6 hydroxydopamine using tyrosine hydroxylase and receptor for brain derived neurotrophic factor  [ **BDNF** ] , trkB  [ **NTRK2** ] , immunohistochemistries.  Ref: 12668152 Exp Neurol, 2003
- As the VMN undergoes sexual differentiation, we compared  **BDNF** , NGF, NT 3, and trkB  [ **NTRK2** ]  mRNA expression in the VMN in males and females at embryonic day 20 and PND 4, but found no differences between them.  Ref: 12824057 Brain Res Mol Brain Res, 2003
- The present study was designed to investigate roles of  **BDNF**  and TrkB  [ **NTRK2** ]  in the expression of methamphetamine induced dopamine release in the nucleus accumbens and dopamine related behaviors induced by methamphetamine in rats.  Ref: 12809697 Neuroscience< MedlineTA>Neuroscience, 2003
- These findings provide further evidence that  **BDNF**  TrkB  [ **NTRK2** ]  pathway is implicated in the methamphetamine induced release of dopamine and the induction of dopamine related behaviors.  Ref: 12809697 Neuroscience< MedlineTA>Neuroscience, 2003
- **BDNF**  in the taste bud cells may act as a neurotrophic factor for innervating sensory neurons through TrkB  [ **NTRK2** ]  receptors of the axons of those neurons, and also may exert autocrine and paracrine trophic actions on neighboring taste bud cells by binding to their TrkB  [ **NTRK2** ]  receptors.  Ref: 12703550 Arch Histol Cytol, 2003
- Genetic as well as pharmacologic deprivation of  **BDNF**  or TrkB  [ **NTRK2** ]  impairs learning and memory.  Ref: 12719654 J Pharmacol Sci, 2003
- These neural precursor cells express the TrkB  [ **NTRK2** ]  as well as the neurotrophin receptor p75, suggesting that they are  **BDNF**  responsive.  Ref: 12589813 Biochem Biophys Res Commun, 2003
- CSC significantly increased the decreased  **BDNF**  and trkB  [ **NTRK2** ]  mRNA in cortex, hippocampus and MBN compared with placebo group.  Ref: 12575383 Zhongguo Zhong Xi Yi Jie He Za Zhi, 2001
- ELISA assay confirmed the presence of  **BDNF**  in the retina, and immunohistochemistry revealed that some tyrosine hydroxylase containing neurons possessed TrkB  [ **NTRK2** ]  receptors.  Ref: 12556415 Invest Ophthalmol Vis Sci, 2003
- In contrast, both the stimulation of dopamine release and induction of dopamine related behaviors by methamphetamine were significantly suppressed by pretreatment with intra nucleus accumbens injection of either  **BDNF**  2.0 & ; mgr ; l rat, 1 100 300 and 1 100 or TrkB  [ **NTRK2** ]  2.0 & ; mgr ; l rat, 1 1000 and 1 100 antibody.  Ref: 12809697 Neuroscience< MedlineTA>Neuroscience, 2003
- In contrast, the expression levels of  **BDNF** , NT 3, trkB  [ **NTRK2** ]  and trkC were significantly higher at the rostral and caudal sites immediately adjacent to the maximal compression site.  Ref: 12774239 Acta Neuropathol Berl , 2003
- Here we investigated the possibility that  **BDNF**  acts through the receptor tyrosine kinase, TrkB  [ **NTRK2** ] , to confer on motor neurons sensitivity to excitotoxic challenge.  Ref: 12614342 J Neurochem, 2003
- Cultured cortical progenitors express  **BDNF**  and NT 3, as well as their receptors TrkB tyrosine kinase  [ **NTRK2** ]  receptor B and TrkC.  Ref: 12832539 J Neurosci, 2003
- These findings suggest that  **BDNF**  TrkB  [ **NTRK2** ]  signaling in the hippocampus plays a crucial role in learning and memory.  Ref: 12719654 J Pharmacol Sci, 2003
- Immunoreactivities for  **BDNF** , NT 3, trkB  [ **NTRK2** ]  and trkC were preferentially localized in the gray matter, particularly in the anterior horn cells.  Ref: 12774239 Acta Neuropathol Berl , 2003
- Here we describe small, dimeric peptides designed to mimic a pair of solvent exposed loops important for the binding and activation of the  **BDNF**  receptor, trkB  [ **NTRK2** ] .  Ref: 12730196 J Biol Chem< MedlineTA>J Biol Chem, 2003
- Glutamate also induced significant changes in glutamate receptor and transporter proteins, as well maintained the association of  **BDNF**  to TrkB  [ **NTRK2** ]  in Müller cells.  Ref: 12654519 Brain Res Mol Brain Res, 2003
- RT PCR showed positive bands at the appropriate sizes for NGF,  **BDNF** , NT3, and NT4, and for the receptors TrkA, TrkB  [ **NTRK2** ] , TrkC, and p75.  Ref: 12882781 Invest Ophthalmol Vis Sci, 2003
- the remaining taste bud cells still expressed  **BDNF**  and TrkB  [ **NTRK2** ] .  Ref: 12703550 Arch Histol Cytol, 2003
- We analyzed eight different neuroblastoma cell lines for the expression of  **BDNF**  and its receptor trkB  [ **NTRK2** ] .  Ref: 12700894 J Cancer Res Clin Oncol< MedlineTA>J Cancer Res Clin Oncol, 2003
- TrkB  [ **NTRK2** ]  IgG and K252a, but not K252b, prevented the  **BDNF**  induced attenuation of the GABA response.  Ref: 12640011 J Physiol< MedlineTA>J Physiol, 2003
- The decreased N methyl D aspartate receptor levels and sustained activation of TrkB  [ **NTRK2** ]  by  **BDNF**  could serve as protective mechanisms for Müller cell survival.  Ref: 12654519 Brain Res Mol Brain Res, 2003
- There is also evidence for activity dependent regulation of the trafficking of the  **BDNF**  receptor, TrkB  [ **NTRK2** ] , including its cell surface expression and ligand induced endocytosis.  Ref: 12663747 Learn Mem< MedlineTA>Learn Mem, 2003
- These results suggest a central role of  **BDNF**  and TrkB  [ **NTRK2** ]  in mediating the neuroprotective effects of this mood stabilizer.  Ref: 12701214 Seishin Shinkeigaku Zasshi, 2003
- Both flat and process bearing ECs were immunopositive for brain derived neurotrophic factor  [ **BDNF** ] , nerve growth factor, neurotrophin 4, and TrkB  [ **NTRK2** ]  but not TrkA.  Ref: 12555206 Glia, 2003
- In the gustatory system, NT3 is unable to replace the actions of  **BDNF**  possibly because of a temporally selective expression of TrkB  [ **NTRK2** ]  in taste neurons.  Ref: 12620975 Development< MedlineTA>Development, 2003
- We found that 1 trk C, a receptor for neurotrophin 3 NT 3, is dominantly expressed in RCEC, but trk B  [ **NTRK2** ] , a receptor for brain derived neurotrophic factor  [ **BDNF** ] , is dominantly expressed in RAEC.  Ref: 12745089 Biochem Biophys Res Commun, 2003
- Furthermore, the basal level of dopamine in the nucleus accumbens was not affected by treatment with both  **BDNF**  and TrkB  [ **NTRK2** ]  antibodies.  Ref: 12809697 Neuroscience< MedlineTA>Neuroscience, 2003
- Using an immunocytochemical method, we examined the expression of  **BDNF**  and its receptor, TrkB  [ **NTRK2** ] , in the taste bud cells of the circumvallate papillae of normal mice and of mice after transection of the glossopharyngeal nerves.  Ref: 12703550 Arch Histol Cytol, 2003
- There was trkB  [ **NTRK2** ]  mRNA but no  **BDNF**  mRNA expression in the macrocyte basal nuclei.  Ref: 12575383 Zhongguo Zhong Xi Yi Jie He Za Zhi, 2001
- Increased neuron losses associated with the presence of truncated TrkB  [ **NTRK2** ]  were most severe in regions where neuron survival is most dependent on brain derived neurotrophic factor  [ **BDNF** ]  and neurotrophin 3.  Ref: 12654337 Neuroscience, 2003
- The results indicate that this stimulated release of 125I  **BDNF**  is not regulated by a feedback mechanism mediated via the TrkB  [ **NTRK2** ]  receptor.  Ref: 12650981 Brain Res< MedlineTA>Brain Res, 2003
- Activation of the TrkB  [ **NTRK2** ]  and the p75NTR by the addition of  **BDNF**  facilitates neuronal commitment and marked neurite genesis.  Ref: 12589813 Biochem Biophys Res Commun, 2003
- METHODS The rat devascularization model was established, and expression of  **BDNF**  mRNA and trkB  [ **NTRK2** ]  mRNA were determined by hybridization in situ method.  Ref: 12575383 Zhongguo Zhong Xi Yi Jie He Za Zhi, 2001
- To test this hypothesis, we compared the ability of LIGA20 and  **BDNF**  to activate TrkB  [ **NTRK2** ] .  Ref: 12829403 Neurotox Res, 2002
- **BDNF**  and its specific receptor TrkB  [ **NTRK2** ]  are concerned with synaptic plasticity as well as maintenance of the nervous system.  Ref: 12706205 J Chem Neuroanat, 2003
- trkB  [ **NTRK2** ]  expression and  **BDNF**  responsiveness in neuroblastoma cells can be induced by all trans retinoic acid RA.  Ref: 12808116 Lab Invest, 2003
- In addition, similar to MC4R mutants, mouse mutants that expresses the  **BDNF**  receptor TrkB  [ **NTRK2** ]  at a quarter of the normal amount showed hyperphagia and excessive weight gain on higher fat diets.  Ref: 12796784 Nat Neurosci< MedlineTA>Nat Neurosci, 2003
- To determine which of the neurotrophins NTs nerve growth factor NGF, brain derived neurotrophic factor  [ **BDNF** ]   **BDNF** , neurotrophin 3 NT3, and neurotrophin 4 5 NT4 and their receptors NTrs, TrkA, TrkB  [ **NTRK2** ] , TrkC, and p75, are present in the adult rat lacrimal gland.  Ref: 12882781 Invest Ophthalmol Vis Sci, 2003
- However, despite overlapping expression of TrkB  [ **NTRK2** ]  and TrkC in many sensory ganglia, brain derived neurotrophic factor  [ **BDNF** ]  and neurotrophin 3 null mutant mice display selective losses in neuronal subpopulations.  Ref: 12620975 Development< MedlineTA>Development, 2003
- For this purpose, serial immunohistochemical and immunoblot analyses were performed for the expression levels of endogenous brain derived neurotrophic factor  [ **BDNF** ]   **BDNF** , neurotrophin NT 3, and their receptors, trkB  [ **NTRK2** ]  and trkC in 24 tip toe walking Yoshimura mice twy twy aged 12 24 weeks.  Ref: 12774239 Acta Neuropathol Berl , 2003
- LTD was induced when the  **BDNF**  TrkB  [ **NTRK2** ]  system was blocked.  Ref: 12736347 J Neurosci, 2003
- RESULTS Expression of  **BDNF**  mRNA and trkB  [ **NTRK2** ]  mRNA reduced obviously in the cerebral cortex and hippocampus of devascularization model, especially in hippocampal CA1 subregion.  Ref: 12575383 Zhongguo Zhong Xi Yi Jie He Za Zhi, 2001
- By 4 weeks after denervation, numerous TrkB  [ **NTRK2** ]  immunoreactive nerve fibers had invaded the papillae, and a few taste buds expressing  **BDNF**  and TrkB  [ **NTRK2** ]  had regenerated.  Ref: 12703550 Arch Histol Cytol, 2003
- We additionally observed the expression of  **BDNF**  and TrkB  [ **NTRK2** ]  in the developing circumvallate papillae of late prenatal and early postnatal mice.  Ref: 12703550 Arch Histol Cytol, 2003
- However, the consequences of  **BDNF**  binding to trkB  [ **NTRK2** ]  receptors in the dorsal horn in relation to pain mechanisms are less well established and are considered in detail.  Ref: 12628355 Trends Pharmacol Sci, 2003
- Here, we show that Fyn is a key molecule linking the  **BDNF**  receptor TrkB  [ **NTRK2** ]  with NMDA receptors, which play an important role in spatial memory formation in a radial arm maze.  Ref: 12663749 Learn Mem< MedlineTA>Learn Mem, 2003
- At P 0, a cluster of  **BDNF**  and TrkB  [ **NTRK2** ]  immunoreactive cells appeared in the dorsal epithelium of the papillae, and was presumed to be primitive taste buds.  Ref: 12703550 Arch Histol Cytol, 2003
- Binding of  **BDNF**  to its cognate receptor TrkB  [ **NTRK2** ]  was also determined using co immunoprecipitation studies.  Ref: 12654519 Brain Res Mol Brain Res, 2003
- In this work the expression and anatomical localization of NGF,  **BDNF**  and NT 3 and of corresponding high affinity receptors TrkA, TrkB  [ **NTRK2** ]  full length and truncated TR isoforms, TrkC and of the low affinity p75 receptor were assessed in surgical samples from adult human lung by reverse transcriptase polymerase chain reaction RT PCR, Western blot and immunohistochemistry.  Ref: 12791675 Am J Respir Cell Mol Biol< MedlineTA>Am J Respir Cell Mol Biol, 2003
- Three proteins mediating signalling pathways are activated by the binding of  **BDNF**  to TrkB  [ **NTRK2** ]  phosphoinositol 3 kinase.  Ref: 12644273 Brain Res, 2003
- Brain derived neurotrophic factor  [ **BDNF** ]  and its receptor, trkB  [ **NTRK2** ] , activate cAMP response element binding protein and the transcription of genes that is crucial for long term potentiation.  Ref: 12689763 Trends Neurosci, 2003
- **BDNF**  treatment of each of the TrkB  [ **NTRK2** ]  expressing cells blocked cisplatin induced cell death.  Ref: 12691830 Cancer Lett, 2003
- Administration of the other TrkB  [ **NTRK2** ]  ligand, brain derived neurotrophic factor  [ **BDNF** ]   **BDNF** , did not affect the normal course of the MyHC isoform transformation in this muscle, indicating that the observed effect is NT 4 5 specific.  Ref: 12771168 J Exp Biol, 2003
- Western blot analysis confirmed these results, showing that the lacrimal gland expresses NGF,  **BDNF** , NT3, and NT4 as well as the NTrs TrkA, TrkB  [ **NTRK2** ] , and TrkC and the p75 protein.  Ref: 12882781 Invest Ophthalmol Vis Sci, 2003
- In contrast, null mutant mice for TrkB  [ **NTRK2** ] , the tyrosine kinase receptor for brain derived neurotrophic factor  [ **BDNF** ]  and neurotrophin 4, displayed a clear loss of locus coeruleus noradrenergic neurons.  Ref: 12810600 Development, 2003
- This contrasts sympathetic neuroblasts, from which neuroblastomas are thought to arise, which neither express trkB  [ **NTRK2** ]  nor are dependent on the p145 trkB  [ **NTRK2** ]  ligands, brain derived neurotrophic factor  [ **BDNF** ]   **BDNF**  or neurotrophin 4 5, for their normal development.  Ref: 12808116 Lab Invest, 2003
- Stimulation of pre synaptic nociceptive afferent fibres induces  **BDNF**  release and consequent activation of TrkB  [ **NTRK2** ]  receptors leading to a post synaptic excitability.  Ref: 12589922 Brain Res Brain Res Rev, 2002
- Double labeling studies showed that  **BDNF**  and NCAM or TrkB  [ **NTRK2** ]  and NCAM colocalized in some of the taste bud cells, but that most taste bud cells were immunopositive for only  **BDNF**  or TrkB  [ **NTRK2** ] .  Ref: 12703550 Arch Histol Cytol, 2003
- Consistent with the presence of trkB  [ **NTRK2** ]  but not trkA,  **BDNF**  causes rapid phosphorylation of MAP kinases ERK1 and ERK2, but NGF does not.  Ref: 12672017 J Neurobiol, 2003
- Messenger RNAs encoding other neurotrophins, including nerve growth factor NGF and neurotrophin 3 NT 3 and the  **BDNF**  receptor trkB  [ **NTRK2** ] , were not selectively localized in neonatal VMN.  Ref: 12824057 Brain Res Mol Brain Res, 2003
